# RTA-408 Protects Kidney from Ischemia-Reperfusion Injury in Mice via Activating Nrf2 and Downstream GSH Biosynthesis Gene

**DOI:** 10.1155/2017/7612182

**Published:** 2017-12-24

**Authors:** Peng Han, Zhiqiang Qin, Jingyuan Tang, Zhen Xu, Ran Li, Xuping Jiang, Chengdi Yang, Qianwei Xing, Xiaokang Qi, Min Tang, Jiexiu Zhang, Baixin Shen, Wei Wang, Chao Qin, Wei Zhang

**Affiliations:** ^1^Department of Urology, The First Affiliated Hospital of Nanjing Medical University, Nanjing 210009, China; ^2^Department of Urology, Jiangsu Province Hospital of TCM, Affiliated Hospital of Nanjing University of TCM, Nanjing 210029, China; ^3^Department of Urology, Yixing People's Hospital, Yixing 214200, China; ^4^Department of Urology, The Second Affiliated Hospital of Nanjing Medical University, Nanjing 210000, China

## Abstract

Acute kidney injury (AKI) induced by ischemia-reperfusion is a critical conundrum in many clinical settings. Here, this study aimed to determine whether and how RTA-408, a novel oleanane triterpenoid, could confer protection against renal ischemia-reperfusion injury (IRI) in male mice. Mice treated with RTA-408 undergoing unilateral ischemia followed by contralateral nephrectomy had improved renal function and histological outcome, as well as decreased apoptosis, ROS production, and oxidative injury marker compared with vehicle-treated mice. Also, we had found that RTA-408 could strengthen the total antioxidant capacity by increasing Nrf2 nuclear translocation and subsequently increased Nrf2 downstream GSH-related antioxidant gene expression and activity. *In vitro* study demonstrated that GSH biosynthesis enzyme GCLc could be an important target of RTA-408. Furthermore, Nrf2-deficient mice treated with RTA-408 had no significant improvement in renal function, histology, ROS production, and GSH-related gene expression. Thus, by upregulating Nrf2 and its downstream antioxidant genes, RTA-408 presents a novel and potential approach to renal IRI prevention and therapy.

## 1. Introduction

Acute kidney injury (AKI) is a global public health concern which impacts approximately 13.3 million patients per year [1]. Ischemia-reperfusion injury (IRI), which presents in numerous clinical conditions including renal transplantation, partial nephrectomy, shock, cardiac surgery, and vascular surgery, is a major cause of AKI in native kidneys, associating with high morbidity, increased Medicare costs, and high mortality (approximately 1.7 million deaths per year) [2, 3]. However, there is no specific therapy to treat or prevent AKI to date. Thus, it is an urgent priority to find an effective treatment [4]. A preclinical animal model may offer us novel underlying pathophysiological mechanisms of AKI and new opportunities for therapeutic intervention in humans.

Oxidative stress (OS) substantially contributes to the pathophysiological process of renal IRI. Renal tubular cell in the outer medulla is very susceptible to OS for its high energy demand and vasculature anatomy [5]. Clinical and experimental studies have shown that the outburst of reactive oxygen species (ROS) production during renal ischemia-reperfusion greatly disequilibrates the redox balance in an injured kidney [6]. Moreover, besides the direct oxidizing effect, ROS can also initiate many other pathophysiological processes of IRI, including neutrophil infiltration [7, 8], apoptosis [9], endoplasmic reticulum stress [10], mitochondrial dysfunction [11], and endothelial activation [12]. Therefore, these factors that can control the ROS detoxification may be potential therapeutic targets.

Nuclear factor-erythroid 2 p45-related factor 2 (Nrf2), a basic leucine zipper redox-sensitive transcription factor, is a master regulator of cell's antioxidant response via binding antioxidant response element (ARE) [13]. Our group has demonstrated that deficiency of Nrf2 caused deteriorated antioxidant capacity in a mouse model of dibutyl phthalate- (DBP-) induced oxidative stress [14]. And we have found that Nrf2 and its downstream target genes were upregulated in injured kidney tissue induced by ischemia and reperfusion (I/R). So we hypothesized that pharmacological activation of Nrf2 would confer protection against IRI.

RTA-408, a newly synthesized oleanane triterpenoid compound, is the most potent known activator of Nrf2. Nowadays, it is investigated in several clinical trials for the therapeutic effect of a variety of diseases including radiation-induced dermatitis, cataract surgery-induced loss of corneal endothelial cells, solid tumors (melanoma and lung cancer), Friedreich's ataxia, and mitochondrial myopathies. Previous *in vitro* studies have shown that RTA-408 had a significant cytoprotective effect via activating the Nrf2 pathway [15–17]. However, the role of RTA-408 in AKI has not been explored to date *in vivo*. In this study, we evaluated the therapeutic effect of RTA-408 *in vivo* and *in vitro* on experimental IRI and tried to elucidate the underlying mechanism.

## 2. Method

### 2.1. Animals and Ethics Statement

Nrf2+/+ mice (8-week-old male C57BL/6J mice, weighting 20–24 g) were bred in Experimental Animals Center of Nanjing Medical University (Nanjing, Jiangsu, China). And Nrf2−/− mice (8-week-old male, weighting 20–23 g) on C57BL/6J gene background were purchased from the Jackson Laboratory (Bar Harbor, Maine, USA). Animals were maintained in our Experimental Animal Center with conventional housing conditions at room temperature (24 ± 2°C), with 50 ± 10% humidity and an automatically controlled 12-hour light/dark cycle under a pathogen-free condition. Besides, these mice were fed a standard rodent chow diet and drinking water during this experiment. They were acclimatized for one week before surgery. The study was carried out in strict accordance with the recommendations in the National Institutes of Health Guide for the Care and Use of Laboratory Animals. All procedures were conducted in experimental animals, and the protocols were approved by the Committee on the Ethics of Animal Research in the Animal Care Facility of Nanjing Medical University (approval number IACUC-1601166).

### 2.2. Mouse Model of Ischemia-Reperfusion Injury

24 h before surgery, mice were intraperitoneally administered with RTA-408 (100 *μ*g/kg body weight; cat. number HY-12212; MCE, USA) or 0.1% dimethyl sulfoxide (DMSO) in PBS as the vehicle. The rationale for RTA-408 dosage was based on the renal function preservation and Nrf2 mRNA activation (Supplementary Figures A and B).

A unilateral ischemia with simultaneous contralateral nephrectomy mouse model was used [18]. Briefly, each mouse was anesthetized by ketamine hydrochloride (100 mg/kg) and xylazine hydrochloride (10 mg/kg) intraperitoneally, and the depth of anesthesia was confirmed by the loss of response to toe pinch. A midline dorsal incision was made, and skin and subcutaneous layers were separated toward both flank areas by blunt dissection. Make a small incision through the right flank muscle and exteriorize the right kidney, cut right kidney, and suture the muscle layer after ligating the right renal pedicle. Make the same procedure on the left side, but using a nontraumatic microvascular clip (Roboz Surgical Instruments, USA) to clamp left renal pedicle for 30 minutes. Mice in the sham group were operated using the same procedure without clamping. During the procedure, a total of 1 ml of warm saline (37°C) was infused into the peritoneal cavity (0.5 ml when the clip was applied and removed) to keep animals well hydrated. The mice were put on a heating pad (38°C) during the procedure until they awoke to keep their body temperatures constant. As soon as the clip was removed, the wounds were sutured and the animals were allowed to recover.

### 2.3. Assessment of Renal Function

The blood samples were obtained from the inferior vena cava at a designated timing point after reperfusion. The blood urea nitrogen (BUN, Urea Assay Kit, cat. number C013-2) and serum creatinine (Scr, Creatinine Assay kit, cat. number C011-2) levels were measured by commercially available assay kits (Jiancheng Bioengineering Institute, Nanjing, China) to assess renal function.

### 2.4. Evaluation of Kidney Histology

The left kidney tissues harvested at 24 h or 96 h postischemia were fixed in 10% buffered formalin, embedded in paraffin, and sectioned in four-micrometer slices. Two experienced pathologists who were blinded to the experimental design scored the histopathological changes in the cortex and outer medulla with periodic acid Schiff (PAS) staining at 24 h, according to the necrotic tubule percentage in five randomly chosen, nonoverlapping high-power fields (HPF) at 400x magnification: 0 (none), 1 (≦10%), 2 (11%–25%), 3 (26%–75%), and 4 (≧76%).

### 2.5. Immunohistochemistry Assessment

To evaluate the magnitude of interstitial neutrophil granulocyte infiltration at 24 h and proliferation at 24 h and 96 h, immunohistochemistry of MPO (GB11224, Wuhan Goodbio Technology, China) and Ki67 (ab16667, Abcam, UK) was performed. And we also quantitatively assessed the number of MPO-positive cells and Ki67-positive cells per high-power fields (HPF) at 400x magnification.

Also, immunohistochemistry of Nrf2 (Cell Signaling Technology, USA) was performed; the result was presented as the number of positive nuclei per high-power fields (HPF) at 400x magnification.

### 2.6. Terminal Uridine Nick-End Labelling (TUNEL) Staining

TUNEL assays were performed on a paraffin-fixed kidney slice using a commercial kit (Roche, Switzerland) according to the manufacturer's instructions. Apoptotic cells had a brown nuclear stain under the microscope and were manually counted in a blinded fashion. The results were presented as the number of TUNEL-positive cells per HPF (400x).

### 2.7. ROS Detection

To detect renal ROS production, an in situ dihydroethidium (DHE, Sigma-Aldrich, USA) fluorescence was performed [19]. Kidney frozen sections (5 *μ*m) were incubated with 1 *μ*mol/l DHE at 37°C in a light-protected, humidified chamber at room temperature for 30 min. The quantitated results of mean density were presented as integrated optical density (IOD) per unit area.

### 2.8. Antioxidant Capacity Assays

Malondialdehyde (MDA), carbonylated protein, total antioxidant capacity (T-AOC), total glutathione (T-GSH), and glutathione to glutathione disulfide (GSH/GSSG) ratio in the supernatant of renal cortical homogenate were estimated by using each assay kit, according to the manufacturer's instructions (Jiancheng Bioengineering Institute, Nanjing, China).

### 2.9. Enzyme Activity Assays

GSH-related enzyme activity was also measured in the supernatant of renal cortical homogenate by a commercially available assay kit (Jiancheng Bioengineering Institute, Nanjing, China).

### 2.10. Western Blotting

A nuclear extract kit (Active Motif, Tokyo, Japan) was used to extract cytoplasmic and nuclear proteins of kidney tissues, according to the manufacturer's protocol. Protein extracts separated upon 7.5% SDS-PAGE were transferred to 0.45 m PVDF membrane (Bio-Rad, California, Hercules, USA). The membranes were incubated with TBST (20 mM Tris-HCL, pH 7.5, 150 mM NaCl, 0.1% Tween 20) with 5% nonfat milk powder for 2 hours before Western blotting overnight at 4°C with rabbit polyclonal antibodies against mouse Nrf2, HO-1, NQO-1, GSR (Cell Signaling Technology, USA), and GCLc (av54576, Sigma, USA). Histone H3 and *β*-actin (Cell Signaling Technology, USA) were used as a protein control to normalize the volume of protein expression. After being washed for 3 × 10 minutes with TBST, the membranes were incubated with HRP-conjugated secondary antibody (1 : 2000, Cell Signaling Technology, USA) for 1.5 hours. Then, after the membranes were washed 3 times with TBST, immunoreactive bands were visualized with electrochemiluminescence reagent (Amersham, Uppsala, Sweden). Densitometric and ImageQuant analyses were subsequently quantified using Image Lab Software (Bio-Rad, USA).

### 2.11. Quantitative Real-Time PCR (qRT-PCR)

Total RNA (2 *μ*g) from mouse kidney tissues or HK-2 cells was reverse transcribed using superscript™ first-strand cDNA synthesis system (Invitrogen, Carlsbad, CA, USA). The reverse transcription was performed at 37°C for 15 min, then 85°C for 5 s. All operations were performed according to the manufacturer's instructions. Real-time PCR (RT-PCR) analyses were performed using StepOnePlus Real-Time PCR system (Applied Biosystems, USA). The qRT-PCR cycle profile was performed at 95°C for10 min, followed by 40 cycles of 15 s at 95°C with a denaturation temperature, 30 s at annealing temperatures of 60°C, and 10 s at 72°C for the final extension. The following primers were used for qRT-PCR:

Nrf2, forward 5′-CTTTAGTCAGCGACAGAAGGAC-3′; reverse 5′-AGGCATCTTGTTTGGGAATGTG-3′

NQO1, forward 5′-AGGATGGGAGGTACTCGAATC-3′; reverse 5′-TGCTAGAGATGACTCGGAAGG-3′

HO-1, forward 5′-AGGTACACATCCAAGCCGAGA-3′; reverse 5′-CATCACCAGCTTAAAGCCTTCT-3′

Gpx2, forward 5′-ATGGCTTACATTGCCAAGTCG-3′; reverse 5′-TGCCTCTGAACGTATTGAAGTC-3′

GCLc, forward 5′-CTACCACGCAGTCAAGGACC-3′; reverse 5′- CCTCCATTCAGTAACAACTGGAC-3′

*β*-Actin, forward 5′-CCTGGCACCCAGCACAAT-3′; reverse 5′-GCTGATCCACATCTGCTGGAA-3′.

*β*-Actin was used as an internal control gene, and the relative mRNA level was calculated using the 2-ΔΔCt method with ABI Step One Software version 2.1. In addition, each experiment was asked to repeat three times.

### 2.12. Cell Culture and Cell Hypoxia/Reoxygenation (H/R) Model

Human renal proximal tubular epithelial cell line (HK-2) was purchased from American Type Culture Collection (ATCC, USA) and cultured in Dulbecco's modified eagle medium (DMEM) (Invitrogen, USA) with nonessential amino acids, 0.05 mg/ml bovine pituitary extract, 50 ng/ml human recombinant epidermal growth factor, 100 units/ml penicillin, 100 *μ*g/ml streptomycin, and 10% fetal bovine serum under 5% CO_2_ and 95% air atmosphere at 37°C.

To establish a cell H/R model, new complete medium was added to 70% confluence cell monolayers after 24 hours of serum starvation and RTA-408 (100 nM) or vehicle incubation. Culture plates were placed in a humidified hypoxic chamber (Thermo Electron) with an atmosphere of 1% O_2_, 94% N_2_, and 5% CO_2_ for 24 hours [20]. After exposure to hypoxia, the medium was refreshed again and the plates were moved to a normoxic cell incubator (21% O_2_ and 5% CO_2_) for 6 hours.

### 2.13. %LDH Release Assay

A LDH microplate titer assay was applied to assess the cell injury as previously described [21]. At the end of various treatments, 100 *μ*l of culture medium was collected to measure media LDH levels. Then, total LDH levels were determined by the addition of Triton X-100 (final concentration 0.1%) to the cells at 37°C for 30 min to release all LDH. The percentage of LDH release was calculated by dividing the media LDH after a treatment by total LDH.

### 2.14. Cell Viability Assay

Cell viability was assessed with Cell Counting Kit-8 (CCK8) (Dojindo Laboratories, Kumamoto, Japan) according to the manufacture's protocol. In general, after different treatments, 10 *μ*l CCK8 solution was added to each plate and cells were incubated for 2 h at 37°C. The cell viability was revealed by the absorbance which was measured at 450 nm.

### 2.15. Immunofluorescent Staining

The localization and expression of GCLc in HK-2 were evaluated by immunofluorescence. HK-2 cells were plated into 12-well plates and cultured until approximately 50% confluence. Then, cells were incubated with or without RTA-408 (100 nM) for24 h and then treated with the H/R procedure. After the specified treatments, cells were fixed for 15 min in 100% methanol at −20°C. After fixation, cells were incubated in 5% blocking serum (1x PBS, 5% goat serum, 0.3% triton-X) for 60 min. Cells were incubated overnight with primary antibodies of GCLc (av54576, Sigma, USA) (1 : 25) in PBS, 1% BSA, and 0.3% triton-X. After an overnight incubation, cells were incubated for 1-2 h in the dark with the fluorochrome-conjugated secondary antibody goat anti-rabbit IgG-FITC (sc-2012, Santa Cruz Biotechnology) (1 : 100) in PBS, 1% BSA, and 0.3% triton-X. Cells were washed, mounted on coverslips with UltraCruz Mounting Medium containing 1.5 mg/ml DAPI (sc-24941, Santa Cruz Biotechnology), and examined using a Nikon Eclipse TE2000-S microscope and X-Cite 120 PC Fluorescence Illumination System (EXFO, Richardson, TX). Images were processed and quantified using ImageJ.

### 2.16. Statistic

Statistical analyses were performed using the analysis of variance (ANOVA), followed by unpaired Student's *t*-test to evaluate the significance of differences between groups. All data are expressed as the mean ± standard deviation (SD) for each group. The differences were evaluated using SPSS 22.0 (Armonk, New York, USA). Each experiment was performed at least 3 times with significant differences accepted when *P* values < 0.05.

## 3. Result

### 3.1. Pretreatment with RTA-408 Improved Renal Function in IRI Mice

**Figure 1:**
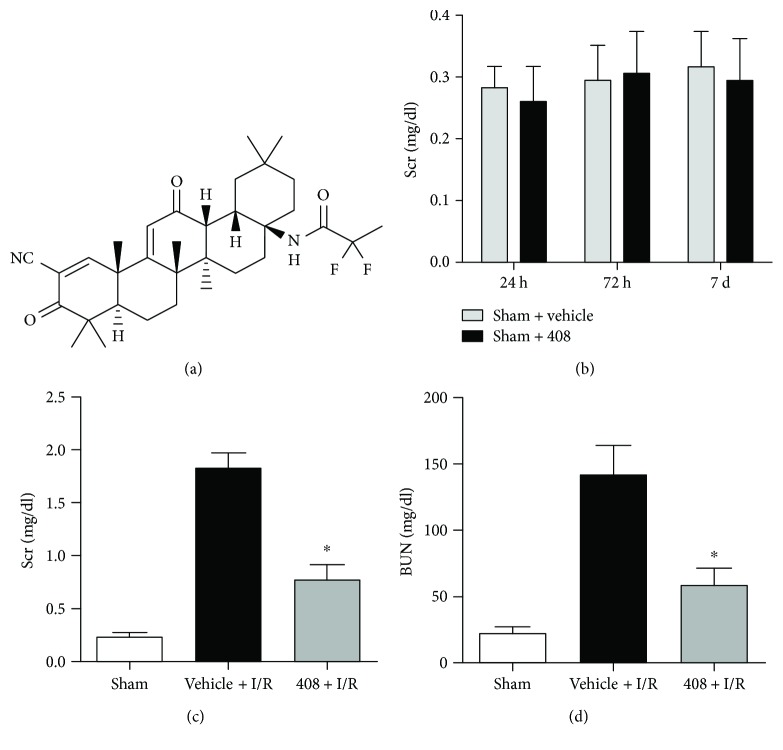
RTA-408 conserved renal function at 24 h reperfusion. (a) Chemical structure of triterpenoid RTA-408. 24 hours before sham or I/R surgery, RTA-408 (100 *μ*g/kg body weight) or vehicle (0.1% DMSO in PBS) was administered intraperitoneally. (b) Serum creatinine level of the sham + vehicle group (*n* = 6) and sham + RTA-408 group (*n* = 6) at 24 h, 72 h, and 7 d reperfusion. (c, d) Serum creatinine and blood urea nitrogen of blood sample collected at 24 h reperfusion from the sham-operated group (sham, *n* = 6), vehicle-treated I/R group (vehicle + I/R, *n* = 11), and RTA-408-treated I/R group (408 + I/R, *n* = 12). Data were presented as mean ± SD. ^∗^Significant difference versus the vehicle + I/R group (Figure 1(c), *P* = 0.002; Figure 1(d), *P* = 0.015).

In order to test whether RTA-408 affects renal function in sham-operated mice, we administered RTA-408 or vehicle to sham-operated mice and evaluated serum creatinine level in a blood sample from tail-cutting. We found that RTA-408 itself had little influence in serum creatinine on sham-operated mice (Figures 1(a) and 1(b)). Nevertheless, in the condition of I/R, RTA-408 significantly improved both serum creatine and BUN when compared with the vehicle + I/R group (Figures 1(c) and 1(d)).

### 3.2. RTA-408 Preserved Renal Histologic Architecture and Mitigates Neutrophil Infiltration

**Figure 2:**
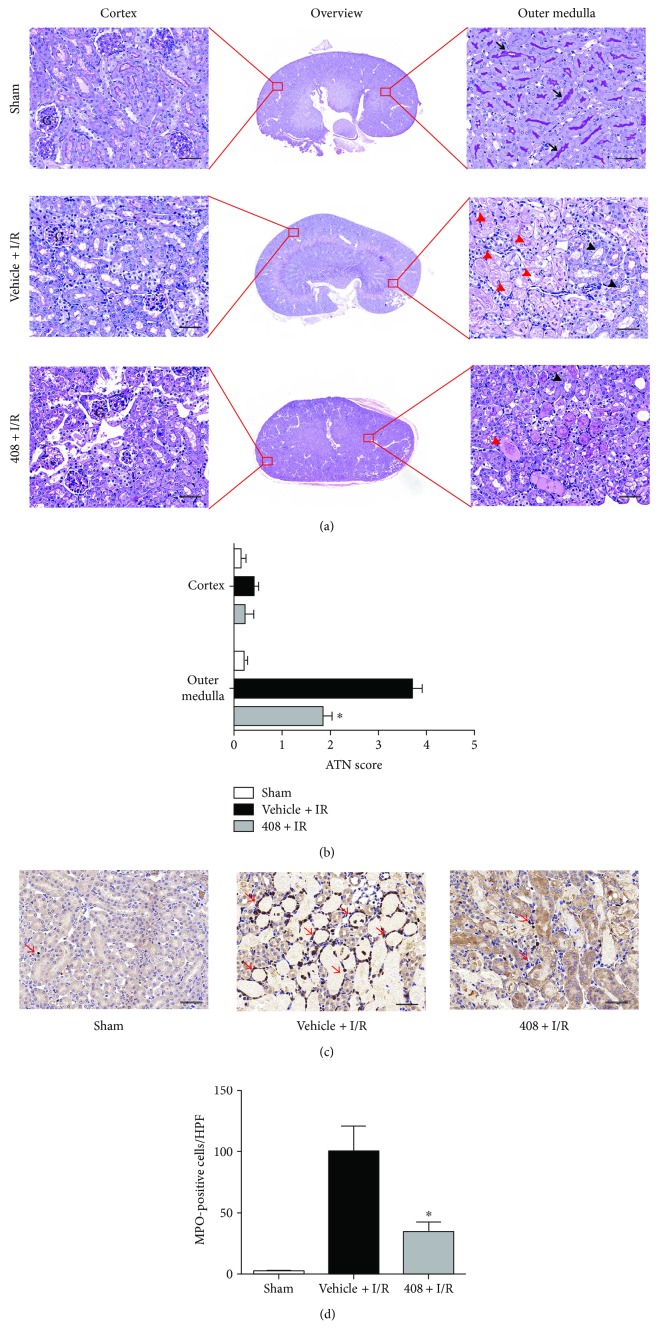
Morphological improvement of RTA-408 on renal ischemia-reperfusion injury. (a) PAS-stained histologic photomicrographs in the indicated area from the sham, vehicle + I/R, and 408 + I/R groups at 24 h reperfusion (*n* = 12). G indicated glomerulus; black arrows indicated brush border; red arrowheads indicated necrotic tubules; and black arrowheads indicated injured tubules. (Magnification 400x; scale bar 50 *μ*m). (b) Quantification of ATN score in the cortex and outer medulla of the above three groups. Data were presented as mean ± SD. ^∗^*P* = 0.021 versus the vehicle + I/R group. (c) Immunohistochemistry was performed to detect MPO-positive cells in kidneys of the above three groups. Red arrows indicate MPO-positive cells. (Magnification 400x; scale bar 50 *μ*m). (d) Quantification of MPO-positive cells per high-power field in different groups. Values are expressed as mean ± SD. ^∗^*P* = 0.019 versus the vehicle + IR group.

To evaluate the tubular injury level, PAS staining was performed at 24 h after IRI. In the cortex area, tissue injury was not as severe as in the outer medulla area. There were seldomly necrotic tubules in the cortex area of both RTA-408-treated and vehicle-treated mice; however, tubular swelling was observed in both groups (G indicated glomerulus). In the outer medulla area of vehicle-treated mice, numerous necrotic tubules (red arrowheads) and injured tubules (black arrowheads) were observed; brush border of tubules was greatly diminished compared to the sham group (black arrows indicated normal brush border). However, these tubular lesions were obviously decreased in the RTA-408-treated mice group (Figure 2(a)). And the representation of histological score indicated a significant difference in the outer medulla area between the 408 + I/R group and the vehicle + I/R group (Figure 2(b)), while there was no significant difference in the cortex area.

In order to measure the neutrophil infiltration into renal interstitial after IRI, MPO assay was performed at 24 h after IRI. MPO-positive cells (red arrows) were greatly increased in the renal interstitial area of the vehicle group compared with the sham group. In contrast, a significant reduction in neutrophil infiltration was observed in the RTA-408-treated mice group compared with the vehicle group (Figure 2(c)). The semiquantitation of the MPO-positive cells was significantly lower in the 408 group compared with the vehicle group at 24 h of reperfusion (Figure 2(d)).

### 3.3. RTA-408 Decreased Renal Tubular Cell Apoptosis and Enhanced Cell Proliferation after IRI

**Figure 3:**
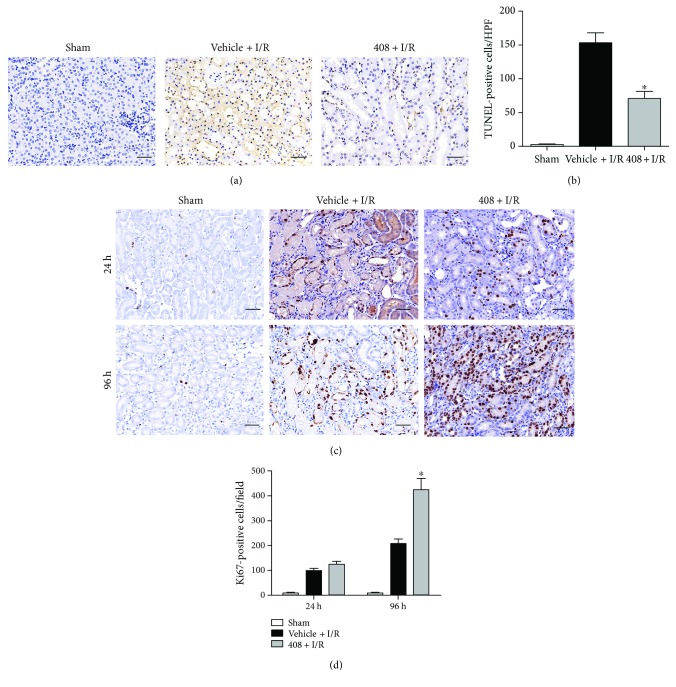
RTA-408's protective role in renal tubule cell apoptosis and proliferation. (a) Representative images (magnification 400x; scale bar 50 *μ*m) of TUNEL immunostaining in the injured kidney at 24 h reperfusion. (b) Quantification of TUNEL-positive cells by an average number of 5 HPF in different groups. Values were expressed as mean ± SD. ^∗^*P* = 0.034 versus the vehicle + I/R group. (c) Representative Ki67 immunostaining result (magnification 400x; scale bar 50 *μ*m) in the injured kidney at different time points after reperfusion. (d) Quantification of Ki67-positive cells by an average number of 5 HPF in different groups. Values were expressed as mean ± SD. ^∗^*P* = 0.027 versus the vehicle + I/R group of 96 h.

Kidneys from RTA-408-treated and vehicle-treated mice were harvested at 24 h and 96 h after IRI. As shown in Figures 3(a) and 3(b), the number of TUNEL-positive cells in the vehicle + I/R group significantly increased at 24 h after IRI compared with those in the sham group, while the kidney slices of the 408 + I/R group represented less TUNEL-positive cells than those of the vehicle + I/R group.

Then, in order to assess whether RTA-408 has an effect on tubular cell proliferation, the Ki67 immunostaining was performed at 24 h and 96 h after IRI. There was no significant difference in the number of Ki67 cells between the vehicle group and the 408 group in the early stage (24 h postischemia); however, at 96 h after surgery, the number of proliferating cells was significantly higher in the 408 + I/R group than in the vehicle + I/R group (Figures 3(c) and 3(d)).

### 3.4. RTA-408 Decreased ROS Production and Tissue Impairment by Enhancing Antioxidant Capacity

**Figure 4:**
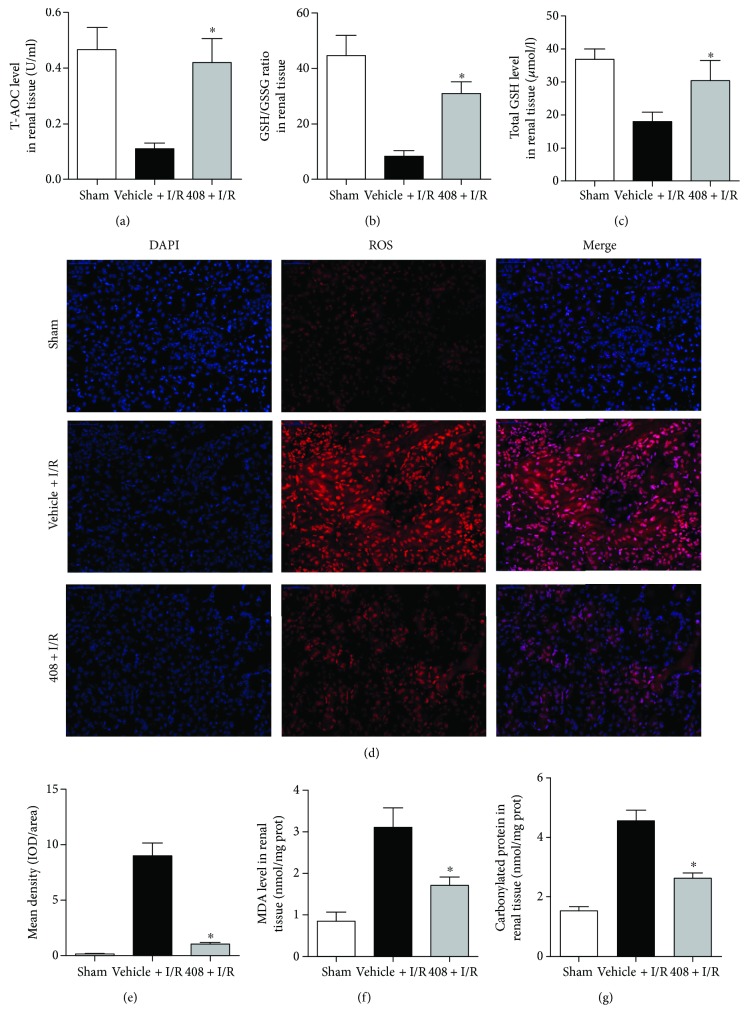
Regulation of kidney antioxidant capacity of RTA-408 on ischemia-reperfusion injury. Quantitative analysis of T-AOC (a) (^∗^*P* = 0.013), GSH/GSSG ratio (b) (^∗^*P* = 0.027), and total GSH (c) (^∗^*P* = 0.015) in injured kidney tissue homogenate at 24 h reperfusion. Representative immunofluorescence image (d) and quantification of ROS production (e) (^∗^*P* = 0.0045) in injured kidney from the sham, vehicle + I/R, and 408 + I/R groups. Lipid peroxidation marker MDA (f) (^∗^*P* = 0.037) and protein peroxidation marker carbonylated protein (g) (^∗^*P* = 0.041) in homogenate renal tissue at 24 h reperfusion. Values were expressed as mean ± SD. ^∗^Significant difference versus the vehicle + I/R group.

The IRI greatly impaired antioxidant capacity and increased the ROS production in the injured kidney at 24 h postischemia. However, RTA-408 administration significantly restored the level of the antioxidant capacity marker, namely, T-AOC, GSH/GSSG ratio, and total GSH (Figures 4(a)–4(c)) in the homogenate of the injured kidney, and diminished the ROS production in the frozen slice of the injured kidney (Figures 4(d) and 4(e)).

Furthermore, the lipid peroxidation marker MDA (Figure 4(f)) and protein peroxidation marker carbonylated protein (Figure 4(g)) were also decreased in the renal tissue homogenate of the 408 + I/R group compared with that of the vehicle + I/R group.

### 3.5. RTA-408 Upregulated Nrf2 and Nrf2 Downstream Gene Expression by Increasing Nrf2 Nuclear Translocation

**Figure 5:**
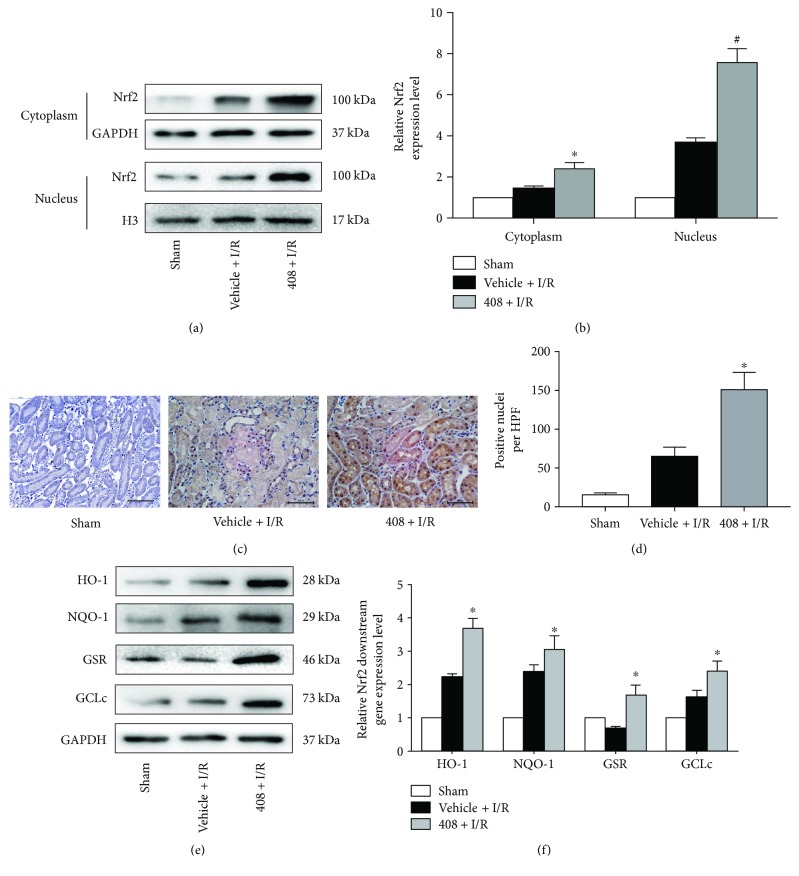
Effect of RTA-408 treatment on Nrf2 and Nrf2 target gene activation. (a, b) Western blot analysis of cytoplasm lysate and nucleus lysate from injured kidney at 24 h reperfusion (^∗^*P* = 0.023; ^#^*P* = 0.011). Nuclear translocation of Nrf2 by RTA-408 was measured by immunohistochemistry staining (^∗^*P* = 0.029). (c, d) Black arrows indicate Nrf2-translocated nuclei (magnification 400x; scale bar 50 *μ*m). The expression intensity quantification was measured by software and shown as a bar graph. (e, f) Western blot analysis showed that Nrf2 downstream genes HO-1 (^∗^*P* = 0.025), NQO-1 (^∗^*P* = 0.037), GSR (^∗^*P* = 0.012), and GCLc (^∗^*P* = 0.027) were notably induced by RTA-408. All data were expressed as mean ± SD. ^∗^^/#^Significant difference versus the vehicle + I/R group.

To explore the mechanism underlying the protective effect of RTA-408, injured kidneys from the sham group, vehicle + I/R group, and 408 + I/R group mice were harvested at 24 h after surgery. Then, the cytoplasm and nucleus protein were extracted, respectively; as Figures 5(a) and 5(b) showed, both the cytoplasm and nucleus Nrf2 expressions were significantly upregulated by the RTA-408 treatment compared to the vehicle + I/R group. Meanwhile, the Nrf2 IHC staining showed that the nuclear translocation in the 408 + I/R group was obviously enhanced compared to that in the vehicle + I/R group (Figures 5(c) and 5(d)). By enhanced Nrf2 nucleus translocation, several Nrf2 downstream ARE genes were greatly upregulated in the protein level. Western blot analysis indicated a significant increase of HO-1, NQO-1, GSR, and GCLc in the 408 + I/R group than in the vehicle + I/R group (Figures 5(e) and 5(f)).

### 3.6. RTA-408 Increased GSH-Related Enzyme Activity

**Figure 6:**
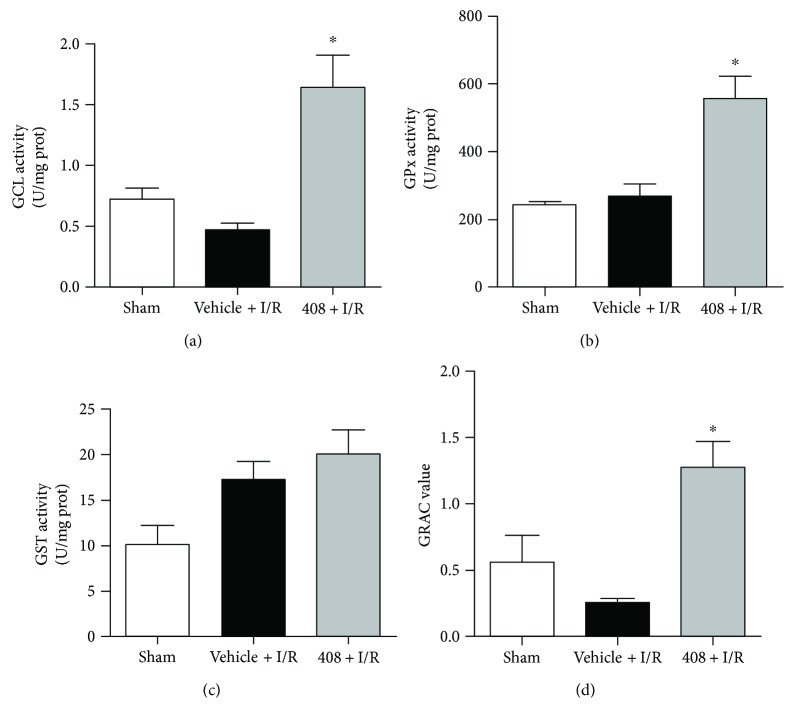
GSH biosynthesis and metabolic enzyme activity were induced by RTA-408. The injured kidney was harvested at 24 h reperfusion, then homogenated, and processed for glutathione-related enzyme activity assay. Glutamyl-cysteine ligase (GCL), glutathione peroxidase (GPx), and glutathione S-transferase (GST) enzyme activities (Figures 6(a)–6(c)) and glutathione reductase activation coefficient (GRAC) value (Figure 6(d)) were measured in the supernatant of renal homogenate following the manufacturer's instructions. Values were expressed as mean ± SD. ^∗^Significant difference versus the vehicle + I/R group (Figure 6(a), *P* = 0.003; Figure 6(b), *P* = 0.015; and Figure 6(d), *P* = 0.011).

To further investigate the effects of RTA-408 on Nrf2 downstream target genes, the GCL activity, GPx activity, GRAC value, and GST activity assay were examined by injured kidney homogenates of the sham group, the vehicle + I/R group, and the 408 + I/R group. As Figures 6(a), 6(b), and 6(d) showed, RTA-408 increased kidney GCL activity, GPx activity, and GRAC value significantly compared with the vehicle. Although there was an increasing trend of GST activity (Figure 6(c)) in the 408 + I/R group, however, there was no significant difference when compared with that in the vehicle + I/R group.

### 3.7. GCL Inhibitor BSO Blunts the Cytoprotective Effect of RTA-408

**Figure 7:**
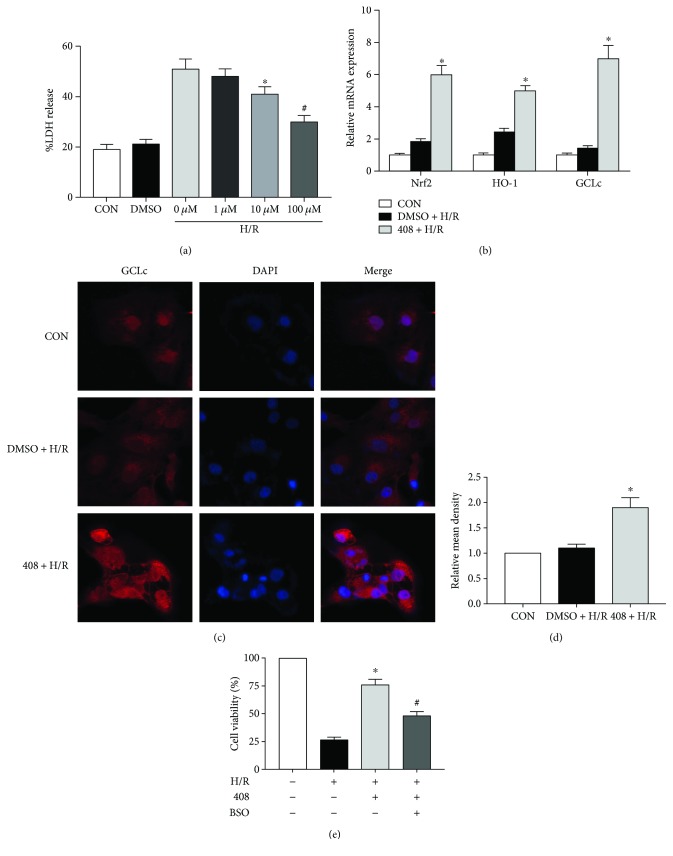
RTA-408 protected HK-2 cells from hypoxia-reoxygenation (H/R) injury, but GCL inhibitor BSO can blunt the protection effect. (a) Dose-dependent protective effect of RTA-408 on H/R-treated HK2 cells. HK2 cells were pretreated with vehicle (DMSO) or RTA-408 (1 nM,10 nM, and 100 nM) for 24 h and were exposed to H/R (24 h hypoxia and 6 h reoxygenation) or not, at which time they were harvested and assessed using a LDH (cell injury marker) assay; each value was expressed as mean ± SD (*n* = 6). ^∗^*P* = 0.025; ^#^*P* = 0.013 versus the 0 *μ*M + H/R group. (b) HK2 cells, pretreated with vehicle (DMSO) or RTA-408 (100 nM) for 24 h, were exposed to H/R. The CON group which did not receive any treatment served as the control. Relative mRNA expression of Nrf2 (^∗^*P* = 0.005), HO-1 (^∗^*P* = 0.013), and GCLc (^∗^*P* = 0.003) was assessed. Values were expressed as mean ± SD. ^∗^ versus the DMSO + H/R group. (c, d) GCLc immunofluorescence in RTA-408- (100 nM) and H/R-treated cells. The quantitated relative mean density of immunofluorescent was expressed as mean ± SD (*n* = 3). ^∗^*P* = 0.035 versus the DMSO + H/R group. (e) GCL inhibitor BSO partly abolished RTA-408's protective effect against H/R. Cell viability was assessed after the different treatment combination of H/R, RTA-408 (100 mM), and L-buthionine sulfoximine (1 mM, BSO, Sigma). Data were expressed as mean ± SD (*n* = 3). ^∗^*P* = 0.016 versus the DMSO + H/R group. ^#^*P* = 0.039 versus the 408 + H/R group.

Thus, we hypothesize that RTA-408 might exert a renoprotective effect via regulating GSH biosynthesis and utilization. In order to further investigate the underlying mechanism, a cell hypoxia/reoxygenation (H/R) model was used. HK-2 cells were cultured to near 80% confluence, and then cells that were pretreated with or without RTA-408 for 24 h were exposed to 24 h hypoxia and 6 h reoxygenation injury, at which time they were harvested and assessed using a LDH assay which severed as a cell injury marker. Hypoxia-reoxygenation significantly increased the %LDH release when compared to the control group. However, RTA-408 pretreatment (10 mM and 100 nM group) resulted in substantial concentration-dependent protection against H/R-induced cytotoxicity (Figure 7(a)). And quantitative real-time PCR confirmed that Nrf2, HO-1, and GCLc were significantly upregulated by RTA-408 pretreatment compared with the vehicle (Figure 7(b)). Immunofluorescent staining also confirmed the increased cellular GCLc expression after treatment with RTA-408 (Figure 7(c)). Next, we evaluated the cell viability when combined with or without GCL inhibitor BSO; as Figure 7(d) showed, when HK-2 cells were preincubated with BSO, the cytoprotective effect of RTA-408 was significantly blunted. The application of BSO confirmed that the cytoprotective effect of RTA-408 was at least partly through the regulation of GSH biosynthesis.

### 3.8. RTA-408 Cannot Protect Nrf2−/− Mice against Renal IRI

**Figure 8:**
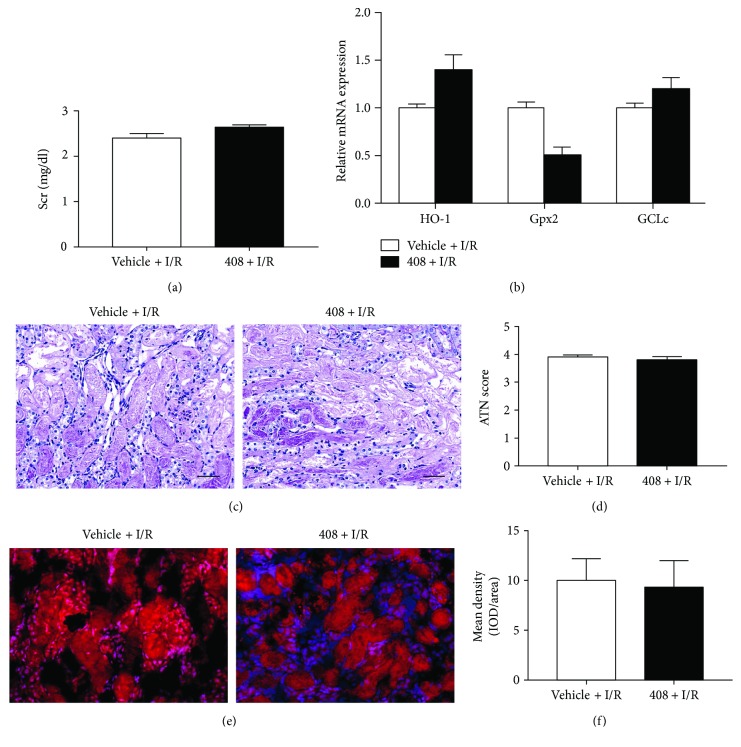
RTA-408 did not confer protection against renal ischemia-reperfusion injury in Nrf2−/− mice. RTA-408 (100 *μ*g/kg bw) or vehicle (0.1% DMSO) was administered intraperitoneally 24 h before surgery. All 8-week-old male Nrf2−/− mice underwent contralateral nephrectomy and 30 min unilateral ischemia followed by reperfusion for up to 24 h. Nrf2−/− mice receiving RTA-408 (*n* = 12) did not show improvement in renal function (a), ATN score (c, d), ROS production (e, f), and Nrf2 downstream gene expression (b) at 24 h reperfusion when compared with Nrf2−/− mice receiving vehicle (*n* = 10). Magnification 400x; scale bar 50 *μ*m.

To confirm that RTA-408 acts dominantly through the Nrf2 pathway, we treated Nrf2-deficient mice with RTA-408 and vehicle to test whether RTA-408 is effective in Nrf2-deficient mice. 8-week-old male Nrf2−/− mice were administered with either RTA-408 or vehicle 24 h before undergoing 30 min unilateral ischemia followed by contralateral nephrectomy and observed up to 24 h. Kidneys from the RTA-408-treated and vehicle-treated Nrf2−/− mice were harvested at 24 h reperfusion and were scored for tissue injury as described previously. RTA-408 did not confer protection in renal function and ATN score in Nrf2−/− mice (Figures 8(a), 8(c), and 8(d)). Also, the ROS production had no difference between the two groups (Figures 8(e) and 8(f)). Harvested kidneys were also analyzed by quantitative real-time PCR for the expression of Nrf2 target genes. RTA-408 did not increase the ischemia-inducible HO1 expression when compared with mice receiving the vehicle and also failed to upregulate Nrf2 target genes (Gpx2 and GCLc) in Nrf2−/− mice (Figure 8(b)), indicating that RTA-408 renoprotection acts predominantly through Nrf2.

## 4. Discussion

Oxidative stress, induced by the pathological overproduction of reactive oxygen species (ROS) and reactive nitrogen species (RNS), plays a substantial role in the development of renal ischemia-reperfusion injury [22]. The outburst of highly electrophilic ROS in the reperfusion process perturbs the balance of renal redox state, which directly causes renal tubular cell damage functionally and structurally by extensive membrane lipid peroxidation, DNA breakdown, and protein inactivation [23]. Furthermore, the excessive ROS can induce inflammasome formation [24], autoimmunity activation [25], and platelet excitation [26], which in turn have both invasive and protective consequences. In addition, researchers validated recently that ROS plays a great role in acute kidney injury (AKI) to chronic kidney disease (CKD) transition by enhancing fibrosis [27]. Naturally, reinforcing the cellular antioxidant defense system to rival oxidative stress is a viable option for IRI treatment.

Nrf2, a cap'n'collar (CNC) basic-region leucine zipper (bZIP) transcription factor, influences intrinsic resistance to oxidative stress by inducing a battery of ROS-detoxifying enzymes and stimulates the production of antioxidants, including the tripeptide glutathione (GSH), thioredoxin (TXN), and sulfiredoxin (SRXN), all of which can reduce oxidized protein thiols [28–30]. A previous study demonstrated that the unbalanced redox state after IRI can be restored by the Nrf2 inducer and kidney injury can be alleviated by enhancing ROS detoxification [31–34]. These data suggest that the Nrf2 pathway is essential in protecting kidney against IRI. However, Nrf2 normally remains a low expression level in the cell because it is rapidly ubiquitinated by Kelch-like ECH-associated protein 1 (Keap1) with consequential degradation via the proteasome.

RTA-408 can reversibly and covalently modify reactive cysteine 151 residues on Keap1, resulting in Keap1 inhibition, thereby activating the Nrf2 signaling pathway and its ARE gene battery to confront ROS insult. In an *in vitro* model [35], RTA-408 can activate the Nrf2 pathway and induce phase II enzyme expression, thereby protecting human retinal pigment epithelial cells from oxidative stress-induced cell apoptosis, preventing ROS over production, and inhibiting protein oxidation. And Reisman et al. [36, 37] reported that topical application of RTA-408 can alleviate radiation-induced dermatitis in mice by a Nrf2-induced cytoprotective response. Although several studies have demonstrated that RTA-408 exerts a strong ROS detoxifying effect via the Nrf2 pathway, our research is the first to show its *in vivo* application in an AKI mouse model.

In the present study, we have found that RTA-408 confers protection against AKI, improving renal function and renal histology, reducing neutrophil infiltration and tubular cell apoptosis, and enhancing tubular cell proliferation at 96 h after IRI. It is likely that these effects were due to the accumulation and nucleus translocation of Nrf2 and induction of downstream cytoprotective genes, including HO-1, NQO1, GSR, and GCLc. The activation of antioxidant genes greatly relieves the ROS outburst induced by IRI and restores the redox state in the injured kidney as indicated by less MDA and carbonylated protein.

In our study, we also have noticed that RTA-408 can restore the total GSH, GSH/GSSG ratio, and T-AOC in injured kidneys. GSH is synthesized in a two-step reaction from cysteine, glutamic acid, and glycine, catalyzed by two rate-limiting enzymes, glutamate-cysteine ligase (GCL) and glutathione synthetase (GSS) [38]. As the most abundant and versatile antioxidant in the cell, GSH takes an active part in scavenging ROS [39], both by direct reaction with ROS and as the cofactor of other antioxidant enzymes [40]. To study the role of RTA-408 in GSH biosynthesis, we evaluated GCLc, the catalytic subunit of GCL, which was greatly induced by RTA-408. To further investigate the role of GCLc in AKI, buthionine sulfoximine (BSO), a GCL inhibitor, was applied in a hypoxia-reoxygenation cell model. When BSO was coincubated, the protective effect of RTA-408 was significantly blunted. It indicates that RTA-408 exerts its cytoprotective effect via upregulating GSH biosynthesis to some extent. And the results of RTA-408 treatment on Nrf2-deficient mice concluded that RTA-408 primarily functions through the Nrf2 pathway, and partly by upregulating GCLc. Overall, these data significantly support our hypothesis that RTA-408 is a potent Nrf2 activator that can confer protection against renal IRI.

Nevertheless, the translation of renal IRI treatment strategy from bench to bedside was not so successful so far and still needed more time and further study. CDDO-Me, a promising Nrf2 inducer which shows great therapeutic potential in a CKD animal model [41–43], was stopped in phase 3 clinical trial for an adverse effect. And the effect of antioxidant supplementation is proved to be limited in the clinical trial of AKI than in an animal model; the reasons are complicated; and the dosage selection, in vivo stability, and severity of AKI can be involved. Excessive amounts of antioxidants or overactivated Nrf2 pathway by exogenous inducer could result in reductive stress which can contribute to cytotoxicity and tissue injury [44, 45]. It will be a new challenge for dose selection to reach a balance point in antioxidant therapy.

**Figure 9:**
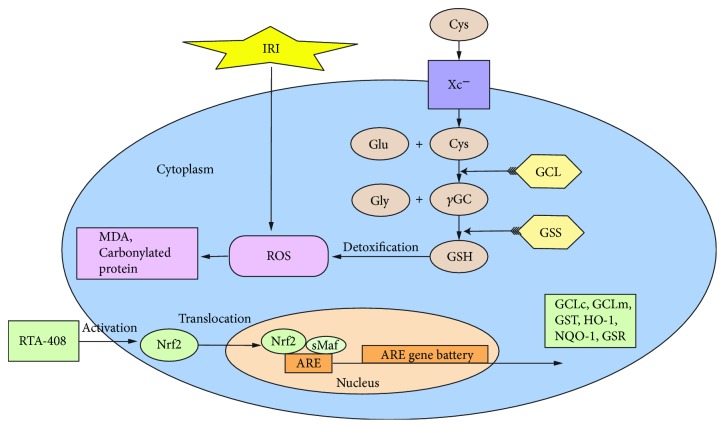
Schematic showing how RTA-408 activates the Nrf2 antioxidant system to protect against renal ischemia-reperfusion injury. Outburst of ROS production during which the I/R damages the lipid and protein in renal tubular cells. RTA-408 is capable of increasing the Nrf2 nucleus translocation and subsequently upregulating of downstream antioxidant genes, including GSH biosynthesis gene GCLc, to protect the kidney against IRI. Cys: cysteine; Gly: glycine; Glu: glutamic acid; *γ*GC: *γ*-glutamylcysteine; GSH: glutathione; Xc^−^: membrane glutamate-cystine transporter; GCLc: glutamate-cysteine ligase catalytic subunit; GCLm: glutamate-cysteine ligase modulate subunit; GSS: glutathione synthetase; GST: glutathione S-transferase; GSR: glutathione reductase; HO-1: heme oxygenase 1; NQO-1: NAD(P)H dehydrogenase 1; ARE: antioxidant response element; MDA: malondialdehyde.

In summary, as Figure 9 showed, our results validated that RTA-408 could induce Nrf2 and downstream antioxidant gene expression, thereby protecting the kidney from renal IRI. The potency and efficacy of RTA-408 in AKI make it an attractive candidate for potential clinical use in treating oxidative stress-related disease. However, it is not sufficient for elucidating the molecular mechanism of RTA-408's renoprotective effect; in our future study, we would investigate whether GCLc knockdown would blunt the renal protective effect of RTA-408 in the renal IRI animal model and HK-2 cell model. And we would further explore RTA-408's antifibrotic effect which may contribute to AKI-CKD-transition. Moreover, we are very interested in whether RTA-408 could inhibit ferroptosis for its strong activation of GSH biosynthesis and Gpx expression.

## Supplementary Materials

Supplementary Materials Figure: Rationale for RTA-408 dosage. To elucidate the relatively suitable RTA-408 dosages for the present study, 24 h before surgery, additional mice were intraperitoneally administered with different dosage of RTA-408 (10 ug/kg body weight, *n* = 3; 100 ug/kg body weight, *n* = 3 and 1000 ug/kg body weight, *n* = 3;), or 0.1% dimethyl sulfoxide (DMSO, *n* = 3) in PBS as vehicle. An unilateral ischemia with simultaneous contralateral nephrectomy mouse model was used. 24 h after surgery, blood samples and injured kidneys were harvested. As showed in figure, RTA-408 can restore the Scr level (A) and activate Nrf2 expression (B) in a dose-dependent manner, however, the dosage of 1000 ug/kg bw showed similar renal function improvement and Nrf2 activation compared to the dosage of 100 ug/kg bw, moreover, higher concentration of the dosage of 1000 ug/kg bw resulted in sedimentation owing to the relatively poor water solubility of RTA-408. Thus, 100 ug/kg body weight dosage of RTA-408 was taken and administered to experimental animals in the current study. ^∗^*P* < 0.05 versus the vehicle + IR group.
