# Supplementary material for: RTA-408 Protects Kidney from Ischemia-Reperfusion Injury in Mice via Activating Nrf2 and Downstream GSH Biosynthesis Gene
Source: Oxid Med Cell Longev. 2017 Dec 24;2017:7612182. doi: 10.1155/2017/7612182 (PMC5757134; doi:10.1155/2017/7612182)
Supplement: Supplementary Materials — Figure: Rationale for RTA-408 dosage. To elucidate the relatively suitable RTA-408 dosages for the present study, 24 h before surgery, additional mice were intraperitoneally administered with different dosage of RTA-408 (10 ug/kg body weight, n = 3; 100 ug/kg body weight, n = 3 and 1000 ug/kg body weight, n = 3;), or 0.1% dimethyl sulfoxide (DMSO, n = 3) in PBS as vehicle. An unilateral ischemia with simultaneous contralateral nephrectomy mouse model was used. 24 h after surgery, blood samples and injured kidneys were harvested. As showed in figure, RTA-408 can restore the Scr level (A) and activate Nrf2 expression (B) in a dose-dependent manner, however, the dosage of 1000 ug/kg bw showed similar renal function improvement and Nrf2 activation compared to the dosage of 100 ug/kg bw, moreover, higher concentration of the dosage of 1000 ug/kg bw resulted in sedimentation owing to the relatively poor water solubility of RTA-408. Thus, 100 ug/kg body weight dosage of RTA-408 was taken and administered to experimental animals in the current study. ∗P < 0.05 versus the vehicle + IR group. [file 7612182.f1.docx]

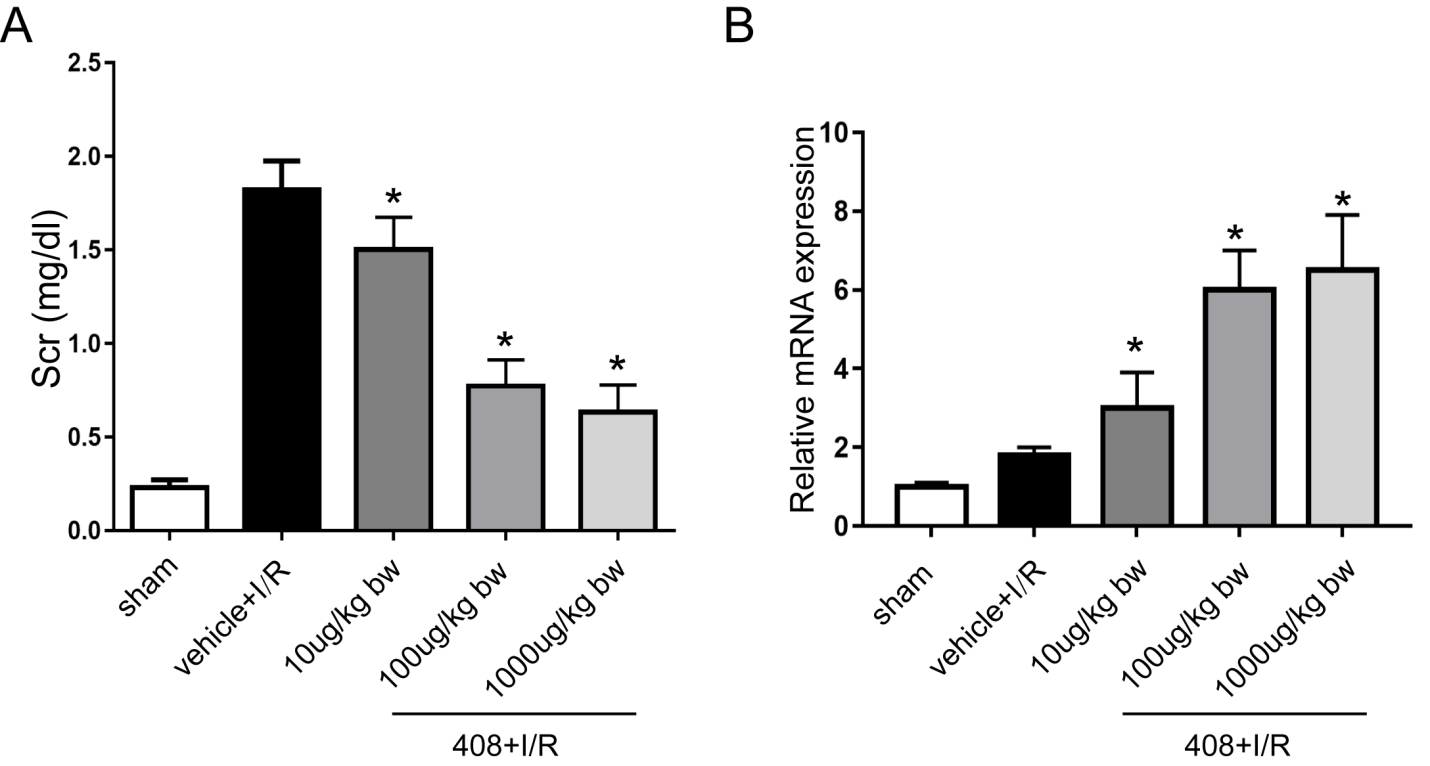


**Figure：Rationale for RTA-408 dosage.**

To elucidate the relatively suitable RTA-408 dosages for the present study, 24h before surgery, additional mice were intraperitoneally administered with different dosage of RTA-408(10ug/kg body weight, n=3; 100ug/kg body weight, n=3 and 1000ug/kg body weight, n=3;), or 0.1% dimethyl sulfoxide(DMSO, n=3) in PBS as vehicle. An unilateral ischemia with simultaneous contralateral nephrectomy mouse model was used. 24h after surgery, blood samples and injured kidneys were harvested. As showed in figure, RTA-408 can restore the Scr level (A) and activate Nrf2 expression (B) in a dose-dependent manner, however, the dosage of 1000ug/kg bw showed similar renal function improvement and Nrf2 activation compared to the dosage of 100ug/kg bw, moreover, higher concentration of the dosage of 1000ug/kg bw resulted in sedimentation owing to the relatively poor water solubility of RTA-408.Thus, 100ug/kg body weight dosage of RTA-408 was taken and administered to experimental animals in the current study. *P<0.05 versus the vehicle+IR group.
